# C1-C2 subluxation in enthesitis-related arthritis: two case reports and literature review of ten cases

**DOI:** 10.1186/s12969-023-00862-3

**Published:** 2023-08-03

**Authors:** Wing Hin Stanford Siu, Chao-Jan Wang, Chieh-Tsai Wu, Chao-Yi Wu, Liang-Shiou Ou

**Affiliations:** 1 School of Medicine, College of Medicine, Chang Gung University, Taoyuan City, Taiwan; 2 Department of Medical Education, Chang Gung Memorial Hospital, Taoyuan City, Taiwan; 3 Department of Medical Imaging and Intervention, Chang Gung Memorial Hospital, Taoyuan City, Taiwan; 4 Department of Neurosurgery, Chang Gung Memorial Hospital, Taoyuan City, Taiwan; 5 Division of Allergy, Asthma and Rheumatology, Department of Pediatrics, Chang Gung Memorial Hospital, 5 Fu-Hsin Street, Kweishan, Taoyuan City Taiwan

**Keywords:** Enthesitis-related arthritis, Juvenile idiopathic arthritis, C1-C2 subluxation, Atlantoaxial subluxation

## Abstract

**Background:**

C1-C2 subluxation is a rare complication of enthesitis-related arthritis (ERA). If left untreated, it may lead to functional impairment or cervical spinal cord compression. This study aims to highlight key points regarding the management of C1-C2 subluxation in ERA.

**Case presentation:**

We present two cases of C1-C2 subluxation: an 8-year-old boy with ERA and 16-year-old boy with ERA with bilateral sacroiliitis. Ten cases of ERA in the literature were reviewed. The diagnosis of C1-C2 subluxation is mostly based on radiographs and cervical spine computed tomography. All patients were treated with non-steroidal anti-inflammatory drugs. Six ERA patients were treated surgically for cervical fusion. Most ERA patients with sacroiliitis had cervical collar protection. Neurologic abnormalities after treatment were not reported. Despite the use of cervical collar, cervical fusion and persisting ankylosis were found in two ERA patients with sacroiliitis without surgical treatment.

**Conclusions:**

Cervical spine protection and ruling out spinal cord compression should be prioritized, in addition to controlling the underlying inflammation in ERA. Cervical halter traction may be applied after severe cervical inflammation is excluded. To reduce the risk of complications, early recognition and appropriate treatments of C1-C2 subluxation in ERA are essential.

**Supplementary Information:**

The online version contains supplementary material available at 10.1186/s12969-023-00862-3.

## Background

Enthesitis-related arthritis (ERA) is a classification of juvenile idiopathic arthritis (JIA) defined by International League of Associations for Rheumatology (ILAR) [1, 2]. The disease mainly involves joints and entheses of lower extremities and may gradually affect the spine or sacroiliac (SI) joints, resembling ankylosing spondylitis [2]. Prior to the introduction of the ILAR classification, those who exhibit peripheral arthritis and enthesitis without axial involvement or radiographic evidence of sacroiliitis may be diagnosed as undifferentiated spondyloarthritis or seronegative enthesopathy and arthopathy (SEA) syndrome [2]. ERA patients, particularly those with axial involvement, generally experience poorer outcomes compared with other JIA subtypes [3]. They often continue to have active disease into adulthood and may eventually develop ankylosing spondylitis [2, 3]. Moreover, early axial involvement can occur, and the progression to ankylosing spondylitis can be unexpected [2, 4]. This highlights the importance of timely identification of axial disease for initiating treatment.

In ankylosing spondylitis, cervical spine can be involved and may progress to C1-C2 subluxation, also known as atlantoaxial subluxation, with a prevalence ranging from 13.8 to 21% in adults [5–8]. However, C1-C2 subluxation is a rare complication in ERA. If left untreated, C1-C2 subluxation may progress to permanent fusion of atlantoaxial joint at an abnormal configuration, potentially leading to compression of spinal cord, compensatory deformity of subaxial cervical spine and functional disability [9]. However, the nonsurgical management approaches of C1-C2 subluxation remain poorly defined and there has been lack of level I evidence comparing different nonsurgical strategies [9]. Therefore, we presented two cases of C1-C2 subluxation in ERA and reviewed similar cases reported in the literature.

### Case presentations

#### Case report of ERA with C1-C2 subluxation

An 8-year-old male without underlying diseases complained of pain in the left heel, left toe, and right proximal interphalangeal joint (PIP) for more than six months. Thus, he walked with a limping gait and was unable to stand steadily. After his classmate collided with his trunk at school about four weeks ago, he started to have difficulty opening his mouth to full extent and head movement was gradually limited to lateral flexion due to cervical stiffness. However, he was able to sit upright from a lying position. There had been no neurological abnormalities, fever, skin rash, or symptoms suggestive of inflammatory back pain or hip pain. He has no family history of spondyloarthritis, inflammatory bowel disease, reactive arthritis, or acute anterior uveitis, except that his uncle has history of ankylosing spondylitis. Therefore, he was referred to us.

On physical examination, modified Schober test, Patrick's test/Flexion, Abduction and External Rotation (FABER) test, Gaenslen’s test, distraction and compression tests of SI joints, thigh thrust test, and straight leg raising test were all negative. However, cervical spine movement was limited to lateral flexion. Additionally, enthesitis of left heel, dactylitis of distal phalange of left foot, and right PIP joint of the ring finger were found during physical examination. Compression and movement of the temporomandibular joint did not elicit pain.

Radiographs of his lumbar-sacral spine and pelvis were unremarkable. However, the radiographs of hand and foot revealed soft tissue swelling in left big toe and right 4th finger, especially around PIP joint. Moreover, the lateral view of cervical-spine radiography revealed an atlantodental interval (ADI) of 4.4 mm. The ADI is also known as the predental space or specified as the anterior atlantodental interval [10]. (Normal ADI on radiograph in children and adults are < 5 mm and < 3 mm, respectively [11]). No associated signs or symptoms of inflammatory bowel disease were found, and ophthalmology consultation concluded absence of uveitis.

His initial lab data indicated an inflammatory status and the human leukocyte antigen (HLA)-B27 was positive (Table 1). Rheumatoid factor (RF), anti-cyclic citrullinated peptides (anti-CCP) antibody and antinuclear antibody (ANA) were all negative. To comprehensively evaluate the inflammatory status of axial skeleton and exclude other inflammatory conditions, (such as osteomyelitis), Gallium-67 inflammation scan was arranged. The result revealed multiple focal regions of increased radioactivity uptake involving the left knee joint, left ankle joint, 1^st^ metatarsophalangeal joint of left foot and 4^th^ PIP joint of right hand.

**Table 1:** Clinical features of enthesitis-related arthritis with C1-C2 subluxation

Patient / [Ref.]	Age (yr) at onset of C1-C2 subluxation / Gender	C1-C2 subluxation onset after initial disease presentation	History of (suspected) trauma	Laboratory examination					Involvement
				**HLA-B27**	**ANA**	**RF**	**CRP (mg/dL)**	**ESR (mm/hr)**	
1 / [12]	9 / Male	> 3 months	None	+	-	-	None	Elevated	Arthritis with synovitis of right knee, left ankle and left elbow; iritis of left eye
2 / [13]	12 / Male	8 months	None	+	- *	-	Normal	75	Left hip, left 5^th^ metatarsal head
3 / [14]	12/ Female	2 years	Previous chiropractic manipulation of cervical spine for pain	+	-	-	None	None	Right knee, left Achilles tendon, right mid-foot small joints, proximal interphalangeal joints
4 / [13]	13 / Male	0 months †	None	+	-	-	None	90	Left knee, left SI joint, both Achilles tendons, left patellar tendon insertions to the patella and tibial tubercle, insertions of the plantar fascia to the calcaneum
5 / [15]	13 / Male	1 year and 1 month	None	+	-	-	1.5	64	Both heels, left elbow
6 / [16]	13 / Male	0 months †	None	+	-	-	None	None	Anterior uveitis with iridolenticular synechiae and band keratopathy
7 / Present patient	8 / Male	6 months	Colliding with a classmate	+	-	-	3.09	116	Left heel, left big toe, right proximal interphalangeal joint of the ring finger
8 / [17]	7 / Female	0 months †	None	-	-	-	Not mentioned at initial disease presentation	30–40	Both knees, both ankles, bilateral SI joints, lumbar spine
9 / [18]	11 / Male	6 months	Subtalar subluxation resulting from a fall	+	-	-	None	55	Both knees, interphalangeal joint of left thumb, left SI joints
10 / [19]	15 / Male	4 years	None	+	-	-	None	None	Both knees, bilateral SI joints, total spine ankylosis
11 / [20]	20 / Male	8 years	None	-	-	-	None	80	Left knee, right Achilles tendon, right plantar fascia insertion, left shoulder, bilateral SI joints, uveitis
12 / Present patient	16 / Male	1 year and 11 months	None	+	-	-	0.46	11	Bilateral SI joints, lumbar spine

HLA-B27, human leukocyte antigen-B27; ANA, antinuclear antibodies; RF, rheumatoid factor; CRP, c-reactive protein; ESR, erythrocyte sedimentation rate; SI joint, sacroiliac joint

^*^Although ANA was weakly detectable in this patient, other rheumatic diseases were not reported other than ERA (which was then under the umbrella of and referred to as “seronegative enthesopathy and arthropathy syndrome” before the introduction of the ILAR classification)

^†^ C1-C2 subluxation was the initial presentation

**Fig. 1:**
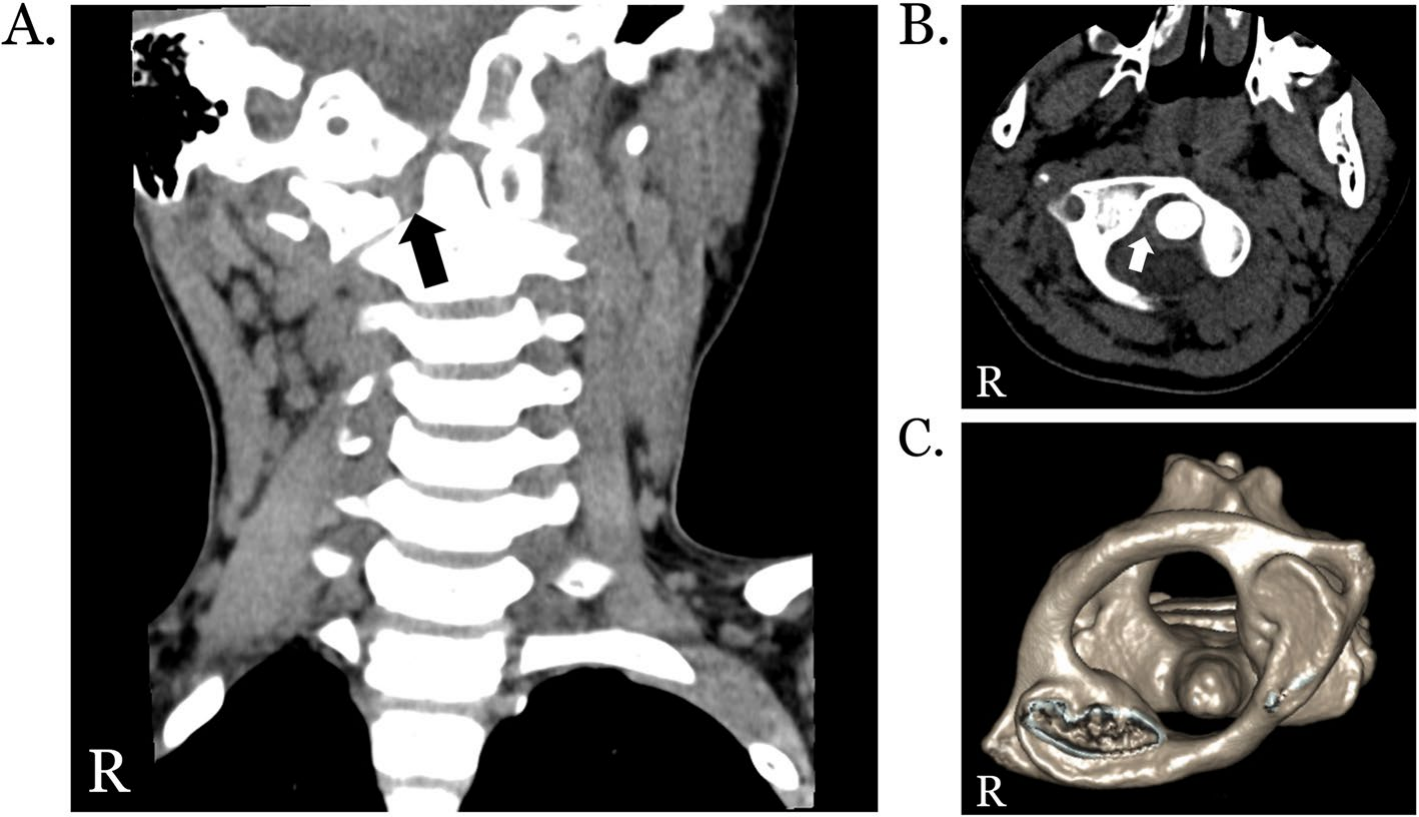
Computed tomography of cervical spine showing C1-C2 subluxation in the present patient with enthesitis-related arthritis. *Cervical spine computed tomography (CT) reveals widened lateral space between the odontoid process and right arch of the atlas in coronal view (black arrow, A) and in axial view (white arrow, B). Cervical spine CT with three-dimensional reconstruction in top-down view (C) depicts rotatory subluxation with asymmetric interspaces between the odontoid process and the arches of the atlas. R: Right*

Under the impression of ERA, he was initially treated with naproxen, sulfasalazine, and methotrexate. However, due to persisting limited range of motion in cervical spine after two days of admission, intravenous methylprednisolone (30 mg/day for four days) was applied. We also arranged computed tomography (CT) of cervical spine, which revealed C1-C2 rotatory subluxation (Fig. 1). Magnetic resonance imaging (MRI) of cervical spine was arranged to evaluate spinal cord compression. Following a negative result, he received cervical collar protection during daytime and halter traction of 1 kg-weight in supine position at night. Two days after methylprednisolone treatment, he was able to open his mouth to a greater extent, clinical improvement was noted in his neck rotation, and the extension and flexion movement. Therefore, we decreased methylprednisolone to 15 mg/day for four days. (His body weight was 24.45 kg). Ten days after admission, we increased the weight of halter traction to 1.5 kg, further provided methocarbamol and switched methylprednisolone to prednisolone (10 mg/day).

Regarding the progress of C1-C2 subluxation, the radiography of cervical spine open mouth anterior–posterior (AP) view of C1-C2 on day 15 of admission indicated improved alignment of C1-C2 when compared to that on day 10. On day 17 of admission, he was discharged with the prescriptions of sulfasalazine, methotrexate, naproxen, prednisolone, methocarbamol, and folic acid. He continued to wear cervical collar in daytime and was treated with halter traction of 1.5 kg weight at night. At the follow-up visit a week after discharge, he was able to open his mouth to a full extent, rotate his head to all directions, and could stand and walk without difficulty. He remained in remission to the time of publication.

### Case report of ERA with bilateral sacroiliitis and C1-C2 subluxation

A 15-year-old male with history of asthma presented to our outpatient clinic complaining of persistent lower back pain with long-standing tilted gesture. The family history was notable for father with ankylosing spondylitis. CRP and ESR were not elevated (Table 1). RF, ANA and anti-CCP antibody were negative. However, HLA-B27 was positive. In imaging studies, the radiography of pelvis frog view showed bilateral sacroiliitis. Additionally, the radiographs of AP and lateral view of lumbar joints and sacroiliac joints both indicated mild scoliosis of lumbar spine, posterior scalloping of the vertebrae, and sclerosis around bilateral SI joints without definite narrowing. Therefore, under the impression of ERA, we started his treatment with naproxen and sulfasalazine.

A year later, he presented at our pediatric neurology outpatient clinic complaining of limited range of motion and pain in his neck for two months. Three months before the visit, he received incision and drainage of a nodulocystic acne with pus in the right preauricular area. Thus, his head maintained a right-turn position for a long time during the procedure. He had no history of trauma or neurological symptoms. On physical examination, the pain exacerbated while rotating his neck and opening his mouth. Neck extension and flexion were normal. However, intermittent numbness of left upper limbs was noted. Otherwise, there were no neurological symptoms and muscle power was full. Additionally, positive modified Schober test revealed impaired lumbar spinal mobility and Gaenslen’s test elicited mild tenderness in the SI joints.

**Fig. 2:**
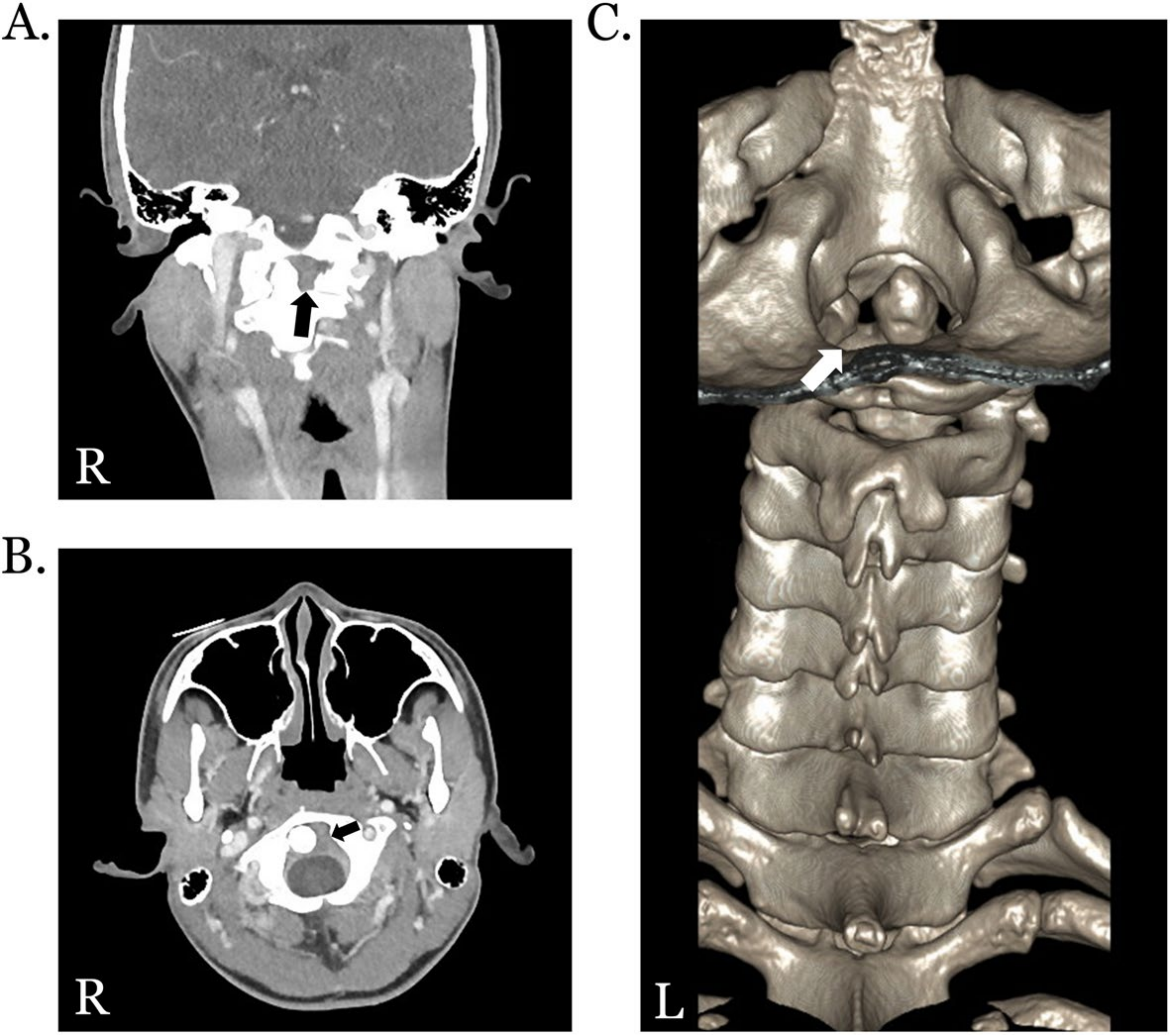
Computed tomography of cervical spine showing C1-C2 subluxation in the present patient with enthesitis-related arthritis with bilateral sacroiliitis. *Computed tomography (CT) of the cervical spine shows widened left interspace between the odontoid process and arches of the atlas in coronal view (black arrow, A) and in axial view (black arrow, B). Three-dimensional reconstruction of cervical spine CT also indicates widened lateral space in the left interspace between the odontoid process and arches of the atlas (white arrow, C). R: Right; L: Left*

In imaging studies, the radiographs of cervical spine in open mouth AP view, and flexion and extension views were unremarkable. However, CT of head and neck with and without enhancement revealed asymmetric lateral space between the C1 arch and the C2 dens (right/left: 1.2/6.8 mm) without anterior or posterior displacement, suggesting rotatory subluxation of C1-C2 (Fig. 2). Additionally, MRI of cervical spine showed asymmetric atlantoaxial space with wider space on the left side, which was consistent with CT findings. There was no cervical cord edema, syrinx formation at the cervical spinal cord, disc protrusion or findings suggestive of radiculopathy or myelopathy.

After admission, we added acetaminophen in addition to naproxen and sulfasalazine. He also received cervical collar protection throughout admission. After eight days of treatment, he was able to rotate his head to the right side 60 degrees away from the midline without eliciting neck pain. Moreover, upper limb numbness subsided. He was discharged with the prescriptions of acetaminophen, naproxen and sulfasalazine, and continued to use cervical collar.

At the follow-up visit about a week later, the range of movement of his neck improved and less pain was noted. The radiograph of cervical-spine open mouth view of C1-C2 still indicated asymmetry of the C1-C2 bilateral dens-lateral mass intervals. However, five months after discharge, the follow-up CT of cervical spine with three-dimensional reconstruction revealed regressive change compared to the previous CT scan during admission. The lateral space between the C1 arch and the C2 dens became less asymmetric, (ie, right/left: 3.7/4.2 mm). He remained in remission to the date of publication.

### Search strategy and results

**Fig. 3:**
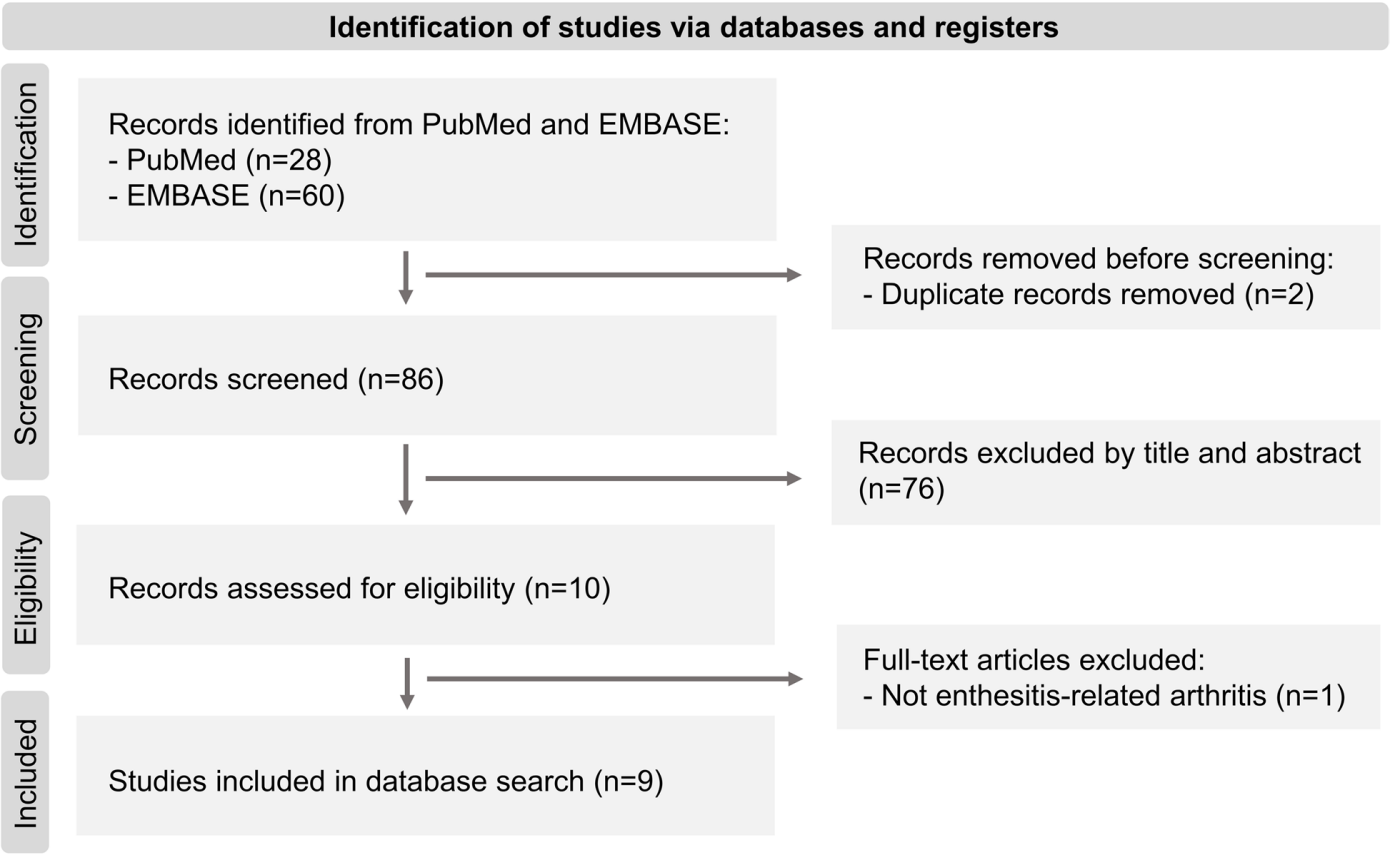
Search strategy for C1-C2 subluxation in enthesitis-related arthritis in the literature

We searched PubMed and Embase up to July 18, 2023, to identify all cases of ERA with C1-C2 subluxation in the literature. We included undifferentiated spondyloarthritis and SEA syndrome in the category of ERA, if the ILAR criteria for ERA were satisfied. We also included patients diagnosed with juvenile ankylosing spondylitis because they would meet the criteria for ERA. No language restrictions were applied. The search strings used were summarized in the electronic supplementary material (Table S1). The process of identification of studies was summarized in Fig. 3. Briefly, we identified a total of ten records from PubMed and EMBASE after removing duplicated records (*n* = 2) and irrelevant records (*n* = 76). We then excluded the article on undifferentiated spondyloarthritis because the case was over 16 years old at onset of symptoms [21]. Finally, we identified ten cases of ERA patients with C1-C2 subluxation from nine records [12–20]. Including our cases, we analyzed a total of twelve patients.

The patients were predominantly male. All patients suffered from C1-C2 subluxation within 2 years, except for two patients. The onset age of C1-C2 subluxation among ERA patients was at least 7 years old. Symptoms of subluxation may include neck pain, limited range of cervical spine movement, torticollis, and numbness in the upper limbs. Clinical presentation of ERA at onset tended to involve pain in extremities. Only three out of twelve patients were notable for their history of suspected trauma. All patients were HLA-B27 positive, except for two patients. CRP at initial disease presentation and ESR were not always measured among the cases included. Out of twelve patients, three had uveitis, and five patients (#8–12) exhibited radiographic changes indicative of sacroiliitis (Table 1).

Most cases reported ADI in radiographic findings. However, not all the cases included findings from CT of the cervical spine and MRI. Six patients were treated with cervical fusion. Most ERA patients with sacroiliitis received cervical collar. Neurologic abnormalities were not reported after treatment. One patient experienced fusion of the cervical spine due to the progression of ankylosing spondylitis, and another patient suffered from persistent ankylosis despite treatments. (Table 2).

**Table 2:** Imaging studies, interventions and outcomes of enthesitis-related arthritis with C1-C2 subluxation

	**Imaging studies**			**Interventions**			**Neurologic signs/symptoms after treatment**	**Range of movement of cervical spine**
**Patient / [Reference]**	**Radiography of ADI**	**CT of cervical spine**	**MRI**	**Medications**	**C1-C2 posterior fusion**	**Cervical collar / traction**		
1 / [12]	> 10 mm	Yes	No	Aspirin, naproxen, ocular steroids	Yes	Cervical collar and Halter traction	None	Fused cervical spine
2 / [13]	4 – 12 mm	Yes	Yes	Diclofenac	Yes	Cervical traction and collar	None	Fused cervical spine
3 / [14]	5 mm*	No	Yes	Naproxen, prednisone, methotrexate, sulfasalazine, etanercept	Yes	None	None	Fused cervical spine
4 / [13]	3 – 8 mm	No	Yes	Tolmetin sodium, sulfasalazine, prednisone	Yes	None	None	Fused cervical spine
5 / [15]	11 mm	No	Yes	NSAID, dexamethasone, methotrexate, sulfasalazine	None	None	None	Improved
6 / [16]	Not measured	Yes	Yes	Methylprednisolone	Yes	None	None	Fused cervical spine
7 / Present patient	4.4 mm	Yes	Yes	Naproxen, methylprednisolone, prednisolone, methotrexate, sulfasalazine, folic acid	None	Cervical collar and Halter traction	None	Improved
8 / [17]	Not measured	No	No	Naproxen, methotrexate, sulfasalazine, etanercept	None	Cervical collar	None	Ankylosis persisted
9 / [18]	8 mm	No	No	Indomethacin	Yes	Cervical collar	None	Fused cervical spine
10 / [19]	11 mm	No	No	Not reported	None	Cervical collar	None	Fused cervical spine
11 / [20]	7 mm	No	No	Not reported	None	None	None	Not reported
12 / Present patient	Not measured	Yes	Yes	Naproxen, sulfasalazine, acetaminophen	None	Cervical collar	None	Improved

NSAID, non-steroidal anti-inflammatory drugs; CT, computed tomography; MRI, magnetic resonance imaging; ADI, atlantodental interval (also known as predental space); *measured from MRI

## Discussion and conclusions

C1-C2 subluxation refers to the partial displacement of C1 and C2 that limits the movement or rotation of the neck. The condition is known by many names, including C1-C2 dislocation, atlantoaxial rotatory subluxation, atlantoaxial rotatory fixation, atlantoaxial dislocation, atlantoaxial displacement, or similar variations [22, 23]. C1-C2 subluxation associated with ERA is rare. In our literature review, there are ten cases of ERA with C1-C2 subluxation. However, if left untreated, C1-C2 subluxation may lead to permanent fusion at abnormal positions, increasing the risk of myelopathy or radiculopathy [9, 22].

### Epidemiology, risk factors and potential pathogenesis

ERA patients are typically male children and adolescents [24–26]. In patients with ERA, inflamed body parts usually involve entheses and joints of the extremities [24–26]. Occasionally, the bilateral SI joints and other axial parts may also be involved [24, 26]. In individuals without predisposing risk factors, C1-C2 subluxation is rare. Atlantoaxial instability is associated with etiologies including trauma, congenital disorders (such as Down syndrome, Morquio syndrome, skeletal dysplasia, congenital osseous abnormalities), inflammatory diseases (such as rheumatoid arthritis), and infections (such as Grisel syndrome) [11, 23]. Among children < 16 years old presented with cervical spine injuries in a multicenter retrospective cohort, the prevalence of atlantoaxial rotatory subluxation was 7% among patients aged < 2 years, 19% among those aged 2–7 years and 7% among those aged 8–15 years [27]. Cervical spine involvement might be associated with underlying autoimmune diseases. For instance, in juvenile idiopathic arthritis (JIA), the prevalence of cervical inflammation could be as high as approximately 60% [28], but the prevalence of radiological cervical spine involvement ranged from 5 to 80% [29]. Notably, atlantoaxial subluxation was the most common cervical spine lesion in polyarticular JIA [29]. However, the epidemiology of C1-C2 subluxation in ERA patients remains unclear. ERA is the most common subtype of JIA in Taiwan and ERA patients with sacroiliitis tend to experience persistent active disease [25, 26]. Since ERA might progress to ankylosing spondylitis, further investigation is needed to determine whether C1-C2 subluxation has a stronger association with ankylosing spondylitis in children compared with ERA (without sacroiliitis).

Among individuals without underlying conditions, trauma-induced C1-C2 subluxation is rare [30–32]. However, in our cases and review, three of the twelve ERA patients had a history of suspected trauma. This might be because children with predisposing factors, such as autoimmune disorders, are susceptible to C1-C2 subluxation with minor trauma [11, 33–35]. This resembles the “deep Koebner effect” in psoriatic arthritis [36], where trauma precipitates the activation of proinflammatory responses involving a “synovio-entheseal complex” [37]. Further investigation is needed to determine if a similar pathogenesis is involved in ERA. Moreover, we suspected that the underlying inflammatory conditions of ERA increased the risk of ligamentous laxity, thereby predisposing these patients to C1-C2 subluxation [11, 35]. Additionally, cervical spine injuries in children are more often found in upper cervical spine (C1-C3) than those in adults [11]. This is because the fulcrum of cervical spine movement in children is at the C2-C3 level, while that of adults is at the C5-C6 level [34]. Collectively, these reasons might explain the association between ERA and C1-C2 subluxation in children. Nevertheless, it is unclear whether C1-C2 subluxation found in children with rheumatic disorders is likely to be triggered by minor trauma. This is probably because minor trauma is seldom recalled by patients.

### Considerations in history taking

In the initial evaluation of ERA patients with suspected C1-C2 subluxation, history taking may consider the possibility of minor trauma, previous upper respiratory infection, post-operative retropharyngeal inflammation (Grisel’s syndrome) as inciting events [11, 38]. Additionally, history taking should consider relevant personal and family history, including congenital disorders (such as Down syndrome, Morquio syndrome, skeletal dysplasia and congenital osseous abnormalities) [11], autoimmune diseases related to ERA (such as ankylosing spondylitis, spondyloarthritis, inflammatory bowel disease, reactive arthritis, or acute anterior uveitis) [1] and inflammatory conditions associated with C1-C2 subluxation (such as rheumatoid arthritis) [39].

Likewise, patients presented with C1-C2 subluxation without known history of ERA may require detailed history taking, assessment of inflammation (such as complete blood count, ESR and CRP levels), and workups for autoimmune disorders. Patients with C1-C2 subluxation might present an opportunity to explore the association between C1-C2 subluxation and ERA.

### Imaging modalities for initial evaluation

**Fig. 4:**
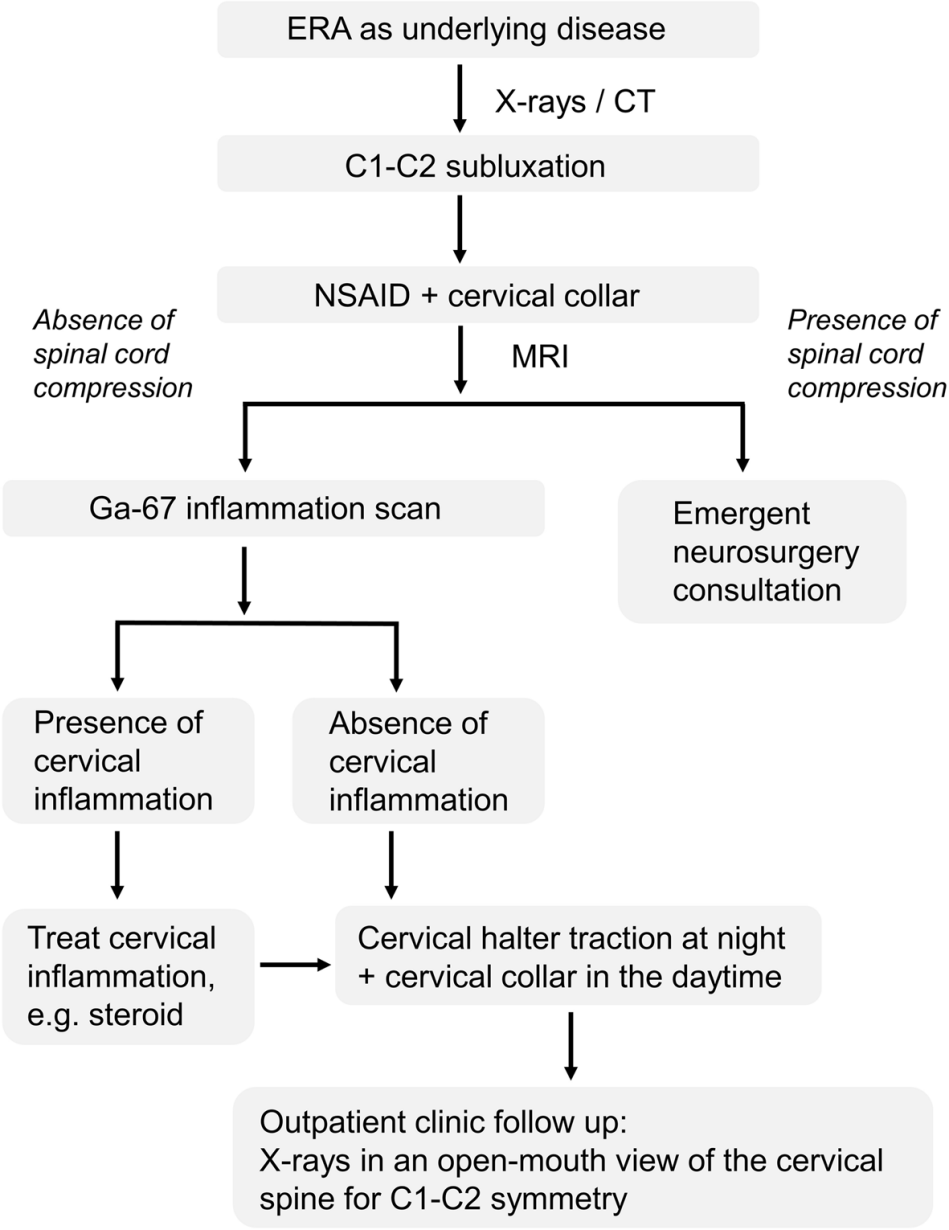
Flowchart of the management of C1-C2 subluxation in enthesitis-related arthritis

We illustrated the flowchart of diagnosis and management of C1-C2 subluxation in ERA patients in Fig. 4. The diagnosis of C1-C2 subluxation can be evaluated by radiographs and CT of cervical spine. In initial evaluation, radiographs may rule out fractures and congenital structural abnormalities. However, in diagnosing atlantoaxial rotatory subluxation among children with acute torticollis, plain radiographs had a sensitivity of 33% and a specificity of 71% when compared to three-dimensional spiral CT, which served as the gold standard [40]. Despite these limitations, open-mouth AP view assesses C1-C2 symmetry [38] and lateral views of cervical spine radiographs allow an estimation of ADI [11]. In children, ADI is typically less than 4–5 mm on radiograph and less than 2.6 mm on CT [10]. The upper normal limit of pediatric ADI might be as low as 3.4–3.5 mm on radiograph [41, 42] and as high as 4.8 mm on CT [43]. It was suggested that ADI exceeding 5 mm indicates instability in children under 8 years old, and exceeding 3 mm indicates instability in older children, as seen in radiographs [44]. Around 20% of children (< 8 years) have an ADI between 3 and 5 mm on a radiograph [44]. However, recent evidence suggests that ADI may be age-independent in children [45]. Nevertheless, the consensus is that an ADI measurement greater than 5 mm on a radiograph may suggest the possibility of atlantoaxial instability [11]. Notably, some of the ADIs of the cases we presented were between 3 and 5 mm, suggesting that the diagnosis of C1-C2 subluxation should not be based on ADI estimated in plain radiographs only. In our experience, atlantoaxial rotatory subluxation may not be obvious in plain radiographs and often did not show ADI more than 5 mm, as in one of our present patients. Moreover, the accuracy of open-mouth AP view may be hindered by limited range of movement of the inflamed temporomandibular joints or head tilting related to torticollis. Therefore, open-mouth views may be unfeasible and can be complemented by lateral views of cervical spine.

Due to the limitations of radiography, cervical spine CT is also essential in the confirmation and classification of atlantoaxial subluxation, particularly when MRI is not readily available [22]. In this study, we used Fielding and Hawkins classification [46]. Other classifications based on dynamic CT scans include Pang and Li classification [47, 48]. 3-position CT with C1-C2 motion analysis was recommended for confirmation and classification of atlantoaxial rotatory subluxation [49]. However, dynamic CT was considered by some investigators to be of poor reliability and reproducibility [50, 51]. Moreover, pediatric patients may not be able to cooperate [50]. Additionally, some studies recommended against CT for routine examination in follow-ups, due to the risks associated with ionizing radiation [22, 52]. Therefore, we recommend using CT for confirmation when cervical radiographs are inconclusive and immediate access to MRI is unavailable (Fig. 4).

After establishing the diagnosis of C1-C2 subluxation, the initial management can be focused on alleviating the cervical inflammation in ERA with NSAID (such as naproxen) and ensuring cervical spine protection (by using cervical collar). The evidence supporting the use of cervical collar and halter for C1-C2 subluxation was discussed elsewhere [9]. Before the use of cervical halter, we should rule out spinal cord compression and severe cervical inflammation. MRI may be used for ruling out conditions of spinal cord compression with transient neurological symptoms or unremarkable neurological examination, especially spinal cord injury without radiographic abnormality (SCIWORA) [53, 54]. If spinal cord compression is found, we suggest consulting neurosurgery service immediately (Fig. 4).

### Cervical protection and conservative management

If there is no spinal cord compression, Gallium-67 inflammation scan may be considered for a comprehensive assessment of axial skeletal inflammation. Additionally, the scan rules out severe cervical inflammation which contradicts the use of cervical halter traction. Cervical inflammation can be controlled by glucocorticoids for short-term treatment to avoid growth disturbance [2]. In our present patients, they did not have severe cervical inflammation that required heavy dosing of anti-inflammatory agents. Therefore, treatments for these patients can follow the ERA guideline recommendations, particularly regarding the use of biologics [55]. Moreover, according to our results, most ERA patients with sacroiliitis had cervical collar protection and no post-treatment neurologic abnormalities were reported in all patients. Despite the use of cervical collar, cervical fusion and persisting ankylosis were found in two ERA patients with sacroiliitis. Notably, various methods of cervical tractions have been reported for the restoration of atlantoaxial alignment, in addition to cervical collar [9, 49]. However, there has been no consistent rationale for each of these methods and no consensus on the duration of halter traction in the literature [9]. We suggest the use of cervical collar in the daytime and halter traction at night for promoting patient compliance after discharge. At outpatient clinic follow-ups, radiographs in open-mouth views may be used for evaluating the restoration of cervical alignment and assessing C1-C2 symmetry (Fig. 4). For recurrent or irreducible atlantoaxial rotatory subluxation, internal fixation and fusion have been recommended [49]. Special attention is necessary for airway management in ERA patients with C1-C2 subluxation due to the risk of life-threatening neurologic injury from worsened subluxation during airway maneuvers, particularly during anesthesia [56].

To decrease the risk of fusion, early recognition and treatment of C1-C2 subluxation in ERA are essential. In our literature review, half of the ERA patients were treated surgically for cervical fusion. An ERA patient had undergone chiropractic neck manipulation before undergoing surgical treatment for cervical fusion, prior to the recognition of subluxation. [14]. Chiropractic neck manipulation is probably unsafe for ERA patients, particularly those experiencing neck symptoms. In fact, atlantoaxial instability is an absolute contraindication of chiropractic neck manipulation [57]. In another case of ERA with sacroiliitis who was treated surgically for cervical fusion, neck pain and stiffness were also not immediately recognized as a possibility of C1-C2 subluxation manifestations [18]. Notably, studies have shown that early recognition and treatment of C1-C2 subluxation were associated with favorable outcomes in terms of higher subluxation reduction and lower recurrences, regardless of treatment modalities [9].

In conclusion, C1-C2 subluxation is a rare association of ERA that may progress to permanent fusion if left untreated. When ERA patients present with torticollis and neck pain, C1-C2 subluxation cannot be ruled out. Since radiographs are limited by low sensitivity, cervical spine CT can be used to confirm the diagnosis of C1-C2 subluxation. Initial management of C1-C2 subluxation in ERA should be focused on protecting the cervical spine and ruling out spinal cord compression. After severe cervical inflammation is ruled out, cervical halter traction can be used to restore cervical alignment. Early recognition and treatment of C1-C2 subluxation in ERA are crucial for minimizing the risk of complications.

### Supplementary Information

**Additional file 1. **

## Data Availability

Not applicable.

## Abbreviations

ERA: Enthesitis-related arthritis
JAS: Juvenile ankylosing spondylitis
ILAR: International League of Associations for Rheumatology
SI: Sacroiliac joints
SEA: Seronegative enthesopathy and arthopathy syndrome
PIP: Proximal interphalangeal joint
FABER: Flexion, Abduction and External Rotation test
CRP: C-reactive protein
ESR: Erythrocyte sedimentation rate
HLA-B27: Human leukocyte antigen
RF: Rheumatoid factor
anti-CCP: Anti-cyclic citrullinated peptides
ANA: Antibody and antinuclear antibody
CT: Computed tomography
AP: Anterior-posterior view
JIA: Juvenile idiopathic arthritis
NSAID: Non-steroidal anti-inflammatory drug
SCIWORA: Spinal cord injury without radiographic abnormality
ADI: Atlantodental interval
