# Supplementary material for: C1-C2 subluxation in enthesitis-related arthritis: two case reports and literature review of ten cases
Source: Pediatr Rheumatol Online J. 2023 Aug 3;21:77. doi: 10.1186/s12969-023-00862-3 (PMC10401742; doi:10.1186/s12969-023-00862-3)
Supplement: Supplementary file 1 — Additional file 1. [file 12969_2023_862_MOESM1_ESM.docx]

**Supplementary material:**

**Table S1**

|  | Search string |
| --- | --- |
| PubMed | (“Atlantoaxial rotatory fixation” OR “Atlantoaxial rotary fixation” OR “Atlanto-axial rotatory fixation” OR “Atlanto-axial rotary fixation” OR “Atlantoaxial rotatory subluxation” OR “Atlantoaxial rotary Subluxation” OR “Atlanto-axial rotatory subluxation” OR “Atlanto-axial rotary subluxation” OR “Atlantoaxial rotatory displacement” OR “Atlantoaxial rotary displacement” OR “Atlanto-axial rotatory displacement” OR “Atlanto-axial rotary displacement” OR “Atlantoaxial fixation” OR “Atlanto-axial fixation” OR “Atlantoaxial subluxation” OR “Atlanto-axial rotatory subluxation” OR “Atlantoaxial displacement” OR “Atlanto-axial displacement” OR “C1-2 subluxation” OR “C1-C2 subluxation” OR “C1/C2 subluxation” OR “C1-2 fixation” OR “C1-C2 fixation” OR “C1/C2 fixation”) AND (“Enthesitis-related arthritis” OR “Juvenile ankylosing spondylitis” OR “juvenile arthritis” OR “juvenile rheumatoid arthritis” OR “juvenile idiopathic arthritis” OR “juvenile chronic arthritis” OR “juvenile spondyloarthropath*” OR “seronegative spondyloarthropath*” OR “seronegative enthesopathy and arthropathy”) |
| EMBASE | (‘Atlantoaxial rotatory fixation’ OR ‘Atlantoaxial rotary fixation’ OR ‘Atlanto-axial rotatory fixation’ OR ‘Atlanto-axial rotary fixation’ OR ‘Atlantoaxial rotatory subluxation’ OR ‘Atlantoaxial rotary Subluxation’ OR ‘Atlanto-axial rotatory subluxation’ OR ‘Atlanto-axial rotary subluxation’ OR ‘Atlantoaxial rotatory displacement’ OR ‘Atlantoaxial rotary displacement’ OR ‘Atlanto-axial rotatory displacement’ OR ‘Atlanto-axial rotary displacement’ OR ‘Atlantoaxial fixation’ OR ‘Atlanto-axial fixation’ OR ‘Atlantoaxial subluxation’ OR ‘Atlanto-axial rotatory subluxation’ OR ‘Atlantoaxial displacement’ OR ‘Atlanto-axial displacement’ OR ‘C1-2 subluxation’ OR ‘C1-C2 subluxation’ OR ‘C1/C2 subluxation’ OR ‘C1-2 fixation’ OR ‘C1-C2 fixation’ OR ‘C1/C2 fixation’) AND (‘Enthesitis-related arthritis’ OR ‘Juvenile ankylosing spondylitis’ OR ‘juvenile arthritis’ OR ‘juvenile rheumatoid arthritis’ OR ‘juvenile idiopathic arthritis’ OR ‘juvenile chronic arthritis’ OR ‘juvenile spondyloarthropath*’ OR ‘seronegative spondyloarthropath*’ OR ‘seronegative enthesopathy and arthropathy’) |
